# Enzyme-Loaded Gel Core Nanostructured Lipid Carriers to Improve Treatment of Lysosomal Storage Diseases: Formulation and In Vitro Cellular Studies of Elosulfase Alfa-Loaded Systems

**DOI:** 10.3390/pharmaceutics11100522

**Published:** 2019-10-11

**Authors:** J. Víctor Álvarez, Carolina Herrero Filgueira, Alexandre de la Fuente González, Cristóbal Colón Mejeras, Andrés Beiras Iglesias, Shunji Tomatsu, José Blanco Méndez, Asteria Luzardo Álvarez, María Luz Couce, Francisco J. Otero Espinar

**Affiliations:** 1 Department of Pharmacology, Pharmacy and Pharmaceutical Technology, School of Pharmacy. Campus Vida, University of Santiago de Compostela, 15872 Santiago de Compostela, Spain; Josevictor.Alvarezgonzalez@nemours.org (J.V.Á.); jose.blanco.mendez@usc.es (J.B.M.); 2 Department of Paediatrics, Hospital Clínico Universitario de Santiago de Compostela, Health Research Institute of Santiago de Compostela (IDIS), CIBERER, MetabERN, 15706 Santiago de Compostela, Spain; cristobal.colon.mejeras@sergas.es; 3 Skeletal Dysplasia Lab Nemours Biomedical Research Nemours/Alfred I. duPont Hospital for Children, 1600 Rockland Road,Wilmington, DE 19803, USA; shunji.tomatsu@nemours.org; 4 Translational Medical Oncology Group (Oncomet), Health Research Institute of Santiago de Compostela (IDIS), University Hospital of Santiago de Compostela, Trav. Choupana s/n, 15706 Santiago de Compostela, Spain; carolina.herrero@rai.usc.es; 5 Nasasbiotech, S.L., Canton Grande 3, 15003 A Coruña, Spain; alexandre.fuente.gonzalez@nasasbiotech.com; 6 Department of Morphological Sciences, School of Medicine, Hospital Clínico Universitario de Santiago de Compostela, 15872 Santiago de Compostela, Spain; andres.beiras@usc.es; 7 Department of Pharmacology, Pharmacy and Pharmaceutical Technology, School of Sciences, Campus de Lugo, University of Santiago de Compostela, 27002 Lugo, Spain

**Keywords:** nanostructured lipid carrier (NLC), lysosomal storage diseases, elosulfase alfa, in vitro cell studies, enzyme activity

## Abstract

Mucopolysaccharidosis IVA (Morquio A) is a rare inherited metabolic disease caused by deficiency of the lysosomal enzyme N-acetylgalatosamine-6-sulfate-sulfatase (GALNS). Until now, treatments employed included hematopoietic stem cell transplantation and enzyme replacement therapy (ERT); the latter being the most commonly used to treat mucopolysaccharidoses, but with serious disadvantages due to rapid degradation and clearance. The purpose of this study was to develop and evaluate the potential of nanostructured lipid carriers (NLCs) by encapsulating elosulfase alfa and preserving its enzyme activity, leading to enhancement of its biological effect in chondrocyte cells. A pegylated elosulfase alfa-loaded NLC was characterized in terms of size, ζ potential, structural lipid composition (DSC and XRD), morphology (TEM microscopy), and stability in human plasma. The final formulation was freeze-dried by selecting the appropriate cryoprotective agent. Viability assays confirmed that NLCs were non-cytotoxic to human fibroblasts. Imaging techniques (confocal and TEM) were used to assess the cellular uptake of NLCs loaded with elosulfase alfa. This study provides evidence that the encapsulated drug exhibits enzyme activity inside the cells. Overall, this study provides a new approach regarding NLCs as a promising delivery system for the encapsulation of elosulfase alfa or other enzymes and the preservation of its activity and stability to be used in enzymatic replacement therapy (ERT).

## 1. Introduction

Mucopolysaccharidoses (MPSs) are a group of inherited lysosomal storage disorders (LSDs) associated with deficiencies in lysosomal enzymes and characterized by the accumulation of glycosaminoglycans (GAGs). MPSs are caused by a deficit of intra-lysosomal specific enzymes or enzymes involved in the transport of proteins from the nucleus to the cytoplasm [1,2]. Morquio A disease (or mucopolysaccharidosis IVA; MPS IVA) [3,4], is caused by the deficiency of lysosomal enzyme N-acetylgalactosamine 6-sulphatase (GALNS, E.C.3.1.6.4) [5], which leads to a progressive accumulation of the substrate of the enzyme at the cellular level in different tissues, such as bone and cartilage [6,7,8,9,10]. GAGs, such as keratan sulfate and chondroitin 6-sulfate, are macromolecules that accumulate at the intracellular level, predominantly in specific tissues [11,12], the extracellular matrix of hyaline cartilage and connective tissues, cardiac valves, the cornea, etc. Currently, the two available therapies for MPS IVA in clinical practice are intravenous administration of the recombinant GALNS enzyme [13,14,15], (elosulfase alfa) to patients weekly (so-called enzyme replacement therapy (ERT)) and hematopoietic stem cell transplantation. ERT with elosulfase alfa is the established treatment for treating somatic symptoms of MPS IVA.

Elosulfase alpha used in ERT is formulated as an aqueous enzyme dispersion in an isotonic, sterile medium for intravenous administration. At present, the main disadvantage of ERT is the difficulty in achieving sufficient concentrations in primary affected tissues (bone and brain), showing a limited impact on bone or neurological manifestations. In fact, to reach therapeutic levels, it is necessary to infuse highly concentrated enzyme solutions slowly for at least three or four hours, in order to achieve distribution in the lysosomes of target tissues. Due to the inefficient biodistribution of infused enzymes to the target site and rapid biodegradation and elimination, the treatment must be repeated after a short period (e.g., weekly). Also, to ensure delivery to lysosomes in the cells of damaged tissues, recombinant enzymes have been commercialized by using the mannose-6-phosphate receptor, which mediates the internalization and delivery of proteins in lysosomes [16,17].

Nevertheless, ERT is commonly associated with several disadvantages. Most ERT based-treatments can produce drug-related hypersensitivity and anaphylactic reactions. As usual in enzyme therapies, patients develop IgG antibodies over time, which can produce immunological problems. Another limitation associated with ERT is related to the inability of the infused enzyme to cross the brain barrier [17,18,19,20]. Furthermore, drug penetration in the avascular cartilage is limited. Overall, conventional ERT results in a lack of improvement regarding neurological and skeletal manifestations. There is no proof that the current ERT used for MPS IVA provides an impact on existing and nonexisting (future) skeletal dysplasia [21,22,23].

In addition, infused enzymes are rapidly cleared from the circulation with a half-life of 2.9 min in mice and 35 min in humans [13,16,17,24]. Therefore, administration of the enzyme must be repeated often an in high doses. Due to the progressive nature of MPS, smaller effects of the drug can lead to life-threating complications. Thus, it is critical to perform enzyme administration under more effective conditions. Although some patients can access ERT by home infusion, the side effects associated with the treatment limit this possibility, therefore, in some cases, patient hospitalization during the infusion of the drug is required [17]. In spite of the rarity of MPS IVA, deteriorating symptoms, progressive morbidity and early mortality, high cost of treatment, and the lack of effective therapies all lead to serious medical, social, and health problems.

As mentioned above, ERT is the current treatment option for MPS IVA. However, there is an unmet challenge regarding the establishment of an effective carrier system to deliver the enzyme to hard-to-reach tissues. Previous reports have indicated a few approaches detailing how carrier systems increase the effect of enzyme delivery and/or diminish adverse effect sof the drug [25,26,27,28]. 

Nanostructured lipid carriers (NLCs) are a second generation of solid lipid nanoparticles (SLNs) that are prepared by blending solid and liquid lipids, leading to an inner amorphous structure which is less ordered and exhibits more stable nanoparticles than conventional SLNs, resulting in higher payload SLNs [29,30,31]. In this work, NLCs were selected as an enzyme delivery system for ERT, taking into consideration its features as a potential carrier system, such as its small size and the lipidic materials which they are composed on, thereby favoring cell internalization. Also, the chemical versatility of NLCs allows the nanoparticles to be retained for a longer period of time in circulation with a reduced possibility of being taken up by macrophages. Compared with other systems, several additional advantages of NLCs are (1) the feasibility of preparation and scale-up, (2) the protection of the enzyme against its degradation or inactivation, and (3) the reduction of associated immunological reactions. NLCs can be also prepared using lipid components, which are recognized as relatively biocompatible and safe for regulatory agencies; therefore, its biocompatibility is ensured [32].

Here, we focused on the design, development, and characterization of stable NLCs for the delivery of elosulfase alpha to lysosomes as a potential therapy for treating LSDs. These NLCs were characterized in vitro in terms of size, surface charge, stability, and cytotoxicity. The biological response of chondrocytes on co-incubation with lipid carriers was also studied. Finally, the biodistribution pattern of elosulfase alfa-loaded NLCs was addressed in vivo with fluorescence imaging.

## 2. Materials and Methods

### 2.1. Materials

Elosulfase alfa (Vimizim^®^) was provided by Biomarin (San Francisco, CA, USA). Block copolymers (Kolliphor^®^ P407 and Kolliphor P188) and d-α-tocopherol polyethylene glycol 1000 succinate were purchased from Sigma Aldrich (St. Louis, MO, USA). Glyceril dibehenate (Compritol 888 ATO) was obtained from Gatefossé (Lyon, France). Trimyristin (Dynasan 114) and triestearin (Dynasan 118) was purchased from IOI Oleo GmbH (Hamburg, Germany). Cholesterol lanolin was obtained from Fluka (Munich, Germany), while olive oil, soy lecitin, and caprylic/capric triglyceride (Miglyol 812N) were obtained from Acofarma (Barcelona, Spain). The water used was ultrapure (milli-Q) and all other chemicals were of analytical grade. 

### 2.2. Methods

#### 2.2.1. Preparation of Elosulfase Alfa-Loaded NLC

The NLC was prepared using the fast-double emulsification (O/W/O) and the low temperature-solidification technique. Briefly, the lipid-forming components of the NLC were dissolved in dichloromethane. Elosulfase alfa dissolution was added to the aqueous phase containing the hydrophilic surfactant (Kolliphor^®^ P407) (Sigma Aldrich) and homogenized using ultrasound (Branson 450) to obtain the first emulsion. A second water phase was added to this resulting O/W emulsion composed of d-α tocopherol polyethylene glycol 1000 in PBS (as a PEGylant agent) (Sigma Aldrich), and the obtained mixture was homogenized again using ultrasound. Finally, Kolliphor^®^ 188 (Sigma Aldrich) solution in PBS was added. The organic solvent was evaporated by rotary evaporation. An optimization study was performed previously to get an optimal formulation in terms of suitable distribution particle size, ζ potential, and physicochemical stability (data not shown). The optimal formula composition of the NLC is displayed in Table 1. As a PEGgylant agent, d-α tocopherol polyethylene glycol 1000 succinate was used (3.30 mM). To investigate the effect of the amount of PEGylant agent on NLC characteristics, different concentrations of d-α tocopherol polyethylene glycol 1000 succinate were used during NLC preparation (0.1, 0.2, and 0.3 mg/mL) whereas the amounts of the rest of the formulation components were kept constant. Afterwards, the particle size, polydispersity index (PdI), and ζ potential were determined. The component of the external phase was Kolliphor^®^ 188, at a 0.5% (*w/v*) with respect to the total formulation. To isolate the NLC, ultracentrifugation (Beckman L8-70M, Ramsey, MN, USA) was used (35 min; 35,000; 15 °C). Finally, each NLC formulation was freeze-dried from an aqueous suspension with different concentrations (5%, 10%, and 20%) of several cryoprotectants (glucose, mannitol, trehalose, and sucrose, from SIGMA-Aldrich, Spain) during 24 h (Telstar Lyoquest-85, Barcelona, Spain) [30,31,33].

#### 2.2.2. Determination of Particle Size and ζ Potential

Particle size distribution (mean diameter of particles and polydispersity index) and ζ potential were determined on a Zetasizer Nano (Malvern Instrument Limited, Worcestershire, UK) equipment. Each NLC formulation was analyzed in ultrapure water at a concentration recommended by the manufacturer, at 25 °C. All measurements were performed in triplicate.

#### 2.2.3. Differential Scanning Calorimetry (DSC)

The thermal properties of the lipids used in the optimized formulation of NLC were determined by differential scanning calorimetry (DSC). Pure solid lipids, pure liquid lipids, and a mixture of solid and liquid lipids were analyzed. The preparation of the lipid samples was similar to the process that the lipids underwent during the NLC preparation. Lipids were dissolved in dichloromethane, and the solvent was then evaporated at 50 °C. Finally, lipids were stored at 24 h at 4 °C to promote the crystallization processes. The measurements were carried out in triplicate, using a DSC TA Discovery, (TA Instruments, New Castle, DE, USA). All experiments were performed under a cooling process from 25 to 0 °C, then samples were heated until 100 °C. Both the cooling and heating processes were performed at a scanning rate of 10 °C/min. The equipment was calibrated to baseline using indium as the standard. The experiments were performed using nonhermetic aluminum pans, in which 1–3 mg of representative samples were weighed and covered with a lid. 

#### 2.2.4. X-ray Diffraction Analysis (XRD)

X-ray diffraction measurements of the powdered samples were performed using a Philips diffractometer (Almelo, The Netherlands) fitted with a Philips PW1710 control unit, a Vertical Philips PW1820/00 goniometer, and an FR590 Enraf Nonius generator. All X-ray powder diffraction patterns were measured with a Philips PW1752/200 curved graphite monochromator and a copper radiation source (λ (Kα1) = 15.406 Å) operating at 40 Kv and 30 mA. The X-ray diffraction pattern was collected by measuring the scintillation response to Cu Kα radiation versus to 2θ value over a 2θ range of 2–50, with a step size of 0.02° and a counting time of 2 s per step. The samples were mounted into a sample holder substrate (Silicon single crystal) to skip the dispersion that could be produced by a glass substrate. Before X-ray analysis, the powdered samples were treated following the same procedure as described above for the DSC measurements.

#### 2.2.5. Transmission Electron Microscopy (TEM)

Elosulfase alfa NLC formulation samples were observed using the JEOL JEM 1011 transmission tlectron ticroscope (TEM; JEOL Inc., Peabody, MA, USA). Two different contrast methods were used to visualize the ultrastructure of the NLC. In the first method, the samples were stained with phosphotungstic acid (2% p/v) and placed in carbon-coated copper grids. In the second method, the NLC as fixed with glutaraldehyde (2.5%) in 0.2 M phosphate buffer overnight. Then, the samples were post-fixed with 1% osmium tetroxide in 0.05 M cacodylate buffer for 1 h and finally embedded in Spurr’s epoxy resin (21). Sections were cut 0.5 microns thick and stained with methylene blue. To observe the inner structure of the NLC, ultrathin sections of 80 Å from the samples of interest were cut and placed to make a 200 hole copper grid, which was stained using the double-contrast method of the ultrathin sections with uranyl acetate and lead citrate. Observations were carried out using a JEOL JEM-1011 microscope (JEOL Ltd., Tokyo, Japan) [34].

#### 2.2.6. Determination of Elosulfase Alfa Activity

To quantify the enzyme activity of elosulfase alfa, an optimized fluorimetric technique routinely used for the enzyme activity in tissues with MPS IVA was used. Elosulfase activity was measured by following the changes in the fluorescence associated with 4-methylumbelliferon (MU-βGal-6S, Moscerdam, Rotterdam) release from galactose-6-sulfate, which was performed in two stages. First, the desulfation on the galactose molecule (position 6) was produced by the addition of enzyme samples to 0.1 M NaCl, 0.02% sodium azide and 5 mM of lead acetate in sodium acetate (0.1 M)/acetic acid (0.1M) buffer, pH 4.3 solution, which was incubated for 18 h for 37 °C. In the second step, the saccharidic residue was released from the 4-methylumbelliferone substrate via a reaction with β-galactosidase (from *Aspergillus orzae*; Sigma-Aldrich) in phosphate-citrate buffer (0.9 M Na_2_HPO_4_/0.9 M NaH_2_PO_4_, pH 4.3). The mixture was incubated at 37 °C for 1 h. The assay was stopped using ethylenediamine (1, 2 diaminethanol) at pH 10 (Sigma-Aldrich). The amount of 4-methylumbelliferone (Sigma-Aldrich) released was determined by fluorescence measurements (FLUOstar OPTIMA (BGM LABTECH Inc. Cary, NC, USA), λ_exc_: 335 nm and λ_em_: 460 nm). The units of elosulfase alfa activity were converted to molar quantities by referring to a calibration curve.

When measuring the enzyme activity of loaded NLCs after co-incubation with chondrocytes, cells were co-incubated at 37 °C for 24 h with free elosulfase alfa and elosulfase alfa-loaded NLC. Then, cells were detached (0.25% trypsin-EDTA 1X, Gibco) and lysed using ultrasound (Bandelin Sonopuls, BANDELIN electronic GmbH & Co. KG, Berlin, Germany). Samples were centrifuged (4 °C for 10 min at 13,000 rpm) and the supernatant was discarded. Protein concentrations in the samples were determined using a protein determination kit based on Bradford (BioRad; Cat #500-0006). Protein samples were frozen at −20 °C for further studies. One unit of enzyme activity of elosulfase alfa was defined as the amount of enzyme that hydrolyzed 1 μmol of substrate per min (or h) at 37 °C per liter of sample (μmol/L/h or nmol/h/mg) [35,36].

#### 2.2.7. Determination of Elosulfase Alfa Loaded in NLC

The enzyme contents of the NLCs were determined after incubation of nanoparticles through Triton X-100 solutions at 37 °C while keeping enzyme functionality. Nanoparticle samples of 200 mg were suspended in 1000 microliters of 15% Triton X-100 solution (Sigma Aldrich). The suspensions were shacked for 15 min and kept at 37 °C (until clear solutions were obtained). The solutions were filtered through a 0.22 µm filter membrane (Millipore, Burlington, MA, USA). Serial dilutions were added to 96 well plates for enzyme activity quantification.

#### 2.2.8. Stability Study of NLCs in Human Plasma

The freeze-dried optimized formulation of the elosulfase alfa-loaded NLC was subjected to stability studies in human plasma, as NLCs are intended for parenteral drug delivery. This assay was performed by incubating the NLC in plasma aliquots from pooled blood samples of 20 healthy patients over different periods of time (0 h, 1 h, 2 h, 3 h, 4 h, 5 h, 24 h, and 48 h) at 37 °C in a 200 mg/mL concentration of NLC. Samples were withdrawn every hour, and NLCs were isolated by ultracentrifugation (14,000 rpm, 30 min, 4 °C) and resuspended in ultrapure water. Then, the particle size, PdI, ζ potential, and/or the enzyme activity remnant were measured.

#### 2.2.9. In Vitro Biological Activity

##### Cell Culture Conditions

The cell line of human chondrocytes, TC28a2, were obtained from Sigma (SCC042; Sigma-Aldrich; Merck) and the pathological fibroblasts of patients with MPS IVA were obtained from international biobanks (biogenic biobank of the Telethon network, http://dppm.galini.org/biobank/). Healthy cells were obtained from the Santiago de Compostela biobank (IDIS) after approval (05/22/2017) of the local Ethics Committee (number 2017/298). Cells were grown in MCCoy’s 5A, supplemented with 10% Fetal bovine serum (FBS) and 1% penicillin-streptomycin (Gibco; Thermo Fischer Scientific, Waltham, MA, USA.), and maintained at 37 °C in the presence of 95% air and 5% CO2 (HERA cell 150, Thermo Fisher Scientific, Lancashire, UK).

##### Primary Chondrocytes from Healthy Patients

Samples of cartilage tissue obtained after traumatology surgery from two donors without metabolic diseases were obtained (healthy patients) after informed consent. The studies consisted of the incubation of the tissue in a culture containing the NLCs with elosulfase alfa at different times and the study of the interaction of NLCs upon co-incubation with chondrocytes using TEM. Cells were extracted from a culture of explants and grown in 25 cm^2^ flasks containing 100,000 cells in MCCoy’s 5A, supplemented with 10% FBS and 1% penicillin-streptomycin (Gibco; Thermo Fischer Scientific, Waltham, MA, USA) and maintained at 37 °C in the presence of 95% air and 5% CO_2_ (HERA cell 150, Thermo Fisher Scientific). At confluence, cell layers were washed with PBS and then scraped and centrifuged (2000 g for 10 min), and the pellet was resuspended and seeded in well plates for further experiments [37].

##### Cytocompatibility Assay 

To determine chondrocyte viability upon co-incubation with NLC, a standard method to measure cytotoxicity, AlarmarBlue^®^ (Thermo Fischer Scientific, Waltham, MA, USA.), was used. In this assay, a redox indicator dye, resazurin, which changes color and fluoresces in response to chemical reduction due to cell growth, evaluated the metabolic activity of cells and in turn, determined the concentration of viable chondrocytes in each well. Fluorescence was detected using Fluostar Optimaequipment (BGM LABTECH Inc. Cary, NC, USA). Cell viability was expressed as the fluorescence measurement with test cells relative to untreated control cells. Primary fibroblasts from healthy patients were seeded in 96 well plates at a density of 4000 cells per well for 24 h at 37 °C. From a stock solution of 500 mg/mL of NLC, 100 µL was used to prepare serial dilutions and was co-incubated with cells for 24 h at 37 °C. After the cells were washed with PBS, 10 µl of AlamarBlue^®^ reagent was added to each well to rech a final volume of 100 µL. Following a 3 h incubation period at 37 °C, the plate was removed from the incubator and the fluorescence was measured with an excitation wavelength of 544 nm and emission wavelength of 590 nm (FLUOstar OPTIMA, BGM LABTECH Inc. Cary, NC, USA).

##### Internalization Studies of NLC in TC28a2 Chondrocytes and Pathological Fibroblasts from MPS IVA Patients 

The capacity of internalization of the optimized NLC containing elosulfase alfa was investigated in the TC28a2 chondrocyte cell line using confocal microscopy and transmission electron microscopy (TEM) and in pathological fibroblasts from Morquio A patients using TEM. Additionally, the enzyme activity of cells after co-incubation with NLCs was studied.

For confocal microscopy, the NLC containing the elosulfase alfa (using different dilutions of NLC formulation from the 200 mg/mL suspension) was stained previously with DiD (1,1′-dioctadecyl-3,3,3′,3′-tetramethylindodicarbocyanine, 4-chlorobenzenesulfonate salt) with the purpose of staining lipids in red; this was then incubated in the co-culture of 100,000 cells in 24 well plates on glass covers at 37 °C or 4 °C for 24 h (HERA cell 150, Thermo Fisher Scientific). After that time, cells were stained with calcein (Fluorexone; Sigma-Aldrich) by incubating cells for 20 min at 37 °C and 4 °C. The co-incubation of NLC in cell cultures were performed at different times, i.e., 1 h, 2 h, or 24 h. Upon NLC exposure, NLC-incorporated cells were fixed for confocal examination using the fluorescence microscope Leica TCS-SP8 (Leica Microsystem, Buffalo Grove, IL, USA).

The cells were imaged using the following resolution conditions: 1024 × 1024 pixels and image size of 184.82 × 184.82 µm. Z-stacks were recorded at 0.3 µm spacing using an objective HC PL APO CS2 63x/1.40 oil, pinhole: 95.3 µm. The cell samples with or without NLC were excited at 638 nm for red dye and 488 nm for green dye, and the emission was filtered through a band-pass filter (646–778 nm and 495–564 nm, respectively).

The interaction of NLC and elosulfase alfa with cells was visualized using TEM with a JEOL JEM 1011 microscope (JEOL Ltd., Tokyo, Japan). TC28a2 chondrocytes or pathological fibroblasts samples were grown onto Thermanox slides (6well plates) and co-incubated for 1 h at 37 °C with NLC (100 µL from 200 mg/mL). After 24 h, cells were centrifuged and fixed with 2.5% of glutaraldehyde in 0.2 M phosphate buffer overnight. The samples were pots-fixed with 1% osmium tetroxide in 0.05 M cacodylate buffer for 1 h and sequentially dehydrated with 50%, 70%, 90%, and 100% methanol. Finally, cells were embedded in Epon 812 resin and sections that were 5 µm thick were cut. Sections were stained with uranyl acetate and lead citrate solution. 

##### Electrophoretic Identification of Elosulfase Alpha in Cells and Quantification by MALDI-TOF Analysis

To analyze the proteins, cells were detached with trypsin, resuspended in the culture medium, centrifuged at 1000 rpm, and washed twice with purified water to remove all remaining proteins. Finally, cells were resuspended in 500 μL of milli-Q water and lysed using ultrasound. Samples were then centrifuged at 13,000 rpm for 10 min at 4 °C to separate the protein extract from the broken cell membranes.

An amount of 100 µg of protein extract as loaded onto a 10% SDS-PAGE gel. The protein band was detected by Sypro-Ruby fluorescent staining (Lonza, Rottenstrass, Switzerland), excised, and processed by manual tryptic digestion. Peptides were extracted by carrying out three 20 min incubations in 40 μL of 60% acetonitrile dissolved in 0.5% HCOOH. The resulting peptide extracts were pooled, concentrated in a SpeedVac, and stored at −20 °C.

Four micrograms of digested peptides was separated using reverse phase chromatography. gradient (micro liquid chromatography system; Eksigent Technologies nanoLC 400, SCIEX, coupled to high-speed Triple TOF 6600 mass spectrometer (ABSciex, Foster City, CA, USA) with a microflow source). The analytical column used for analysis was the silica-based reversed-phase column Chrom XP C18 150 × 0.30 mm with 3 mm particle size and 120 Å pore size (Eksigent, ABSciex, Woodlands Central Indus. Estate, Singapore). The trap column was the YMC-TRIART C18 (YMC Technologies, Teknokroma, Barcelona, Spain) with a 3 mm particle size and 120 Å pore size, switched online with the analytical column. The loading pump delivered a solution of 0.1% formic acid in water at 10 µL/min. The micro-pump generated a flow-rate of 5 µL/min and was operated under gradient elution conditions, using 0.1% formic acid in water as mobile phase A and 0.1% formic acid in acetonitrile as mobile phase B. The peptides were separated using a 90 min gradient ranging from 2% to 90% mobile phase B (mobile phase A: 2% acetonitrile, 0.1% formic acid; mobile phase B: 100% acetonitrile, 0.1% formic acid). 

Data acquisition was performed using the TripleTOF 6600 System (SCIEX, Foster City, CA, USA) via a data-dependent workflow. Source and interface conditions were an ion spray voltage floating (ISVF) of 5500 V, curtain gas (CUR) 25, collision energy (CE) 10, and ion source gas 1 (GS1) 25. The instrument was operated with Analyst TF 1.7.1 software (ABSciex, Woodlands Central Indus. Estate, Singapore). The switching criteria was set to ions greater than a mass-to-charge ratio (m/z) of 350 and smaller than m/z of 1400 with a charge state of 2–5, a mass tolerance of 250 ppm, and an abundance threshold of more than 200 counts (cps). Former target ions were excluded for 15 s. The instrument was automatically calibrated every 4 h using external calibration tryptic peptides from PepCalMix.

Pathological fibroblasts were incubated with chondroitin-6-sulphate (C6S)(Sigma Aldrich), allowing for the quantification of GAGs. 

For the quantification of GAG concentrations in cells, 1-9 dimethylmethylene blue (DMB) (Sigma Aldrich) was used. 

Pathological fibroblasts, with a total of 100,000 cells, were seeded in well plates and left for 24 h to adhere to the plate surface. Cells were incubated with a concentration of 6.25 mg/dL of C6S to promote the intracellular accumulation. Tree replicates were incubated additionally with 100 μL of solution containing elosulfase alfa-NLC at a concentration of 200 mg/mL (equivalent to 50 ng/mL of free enzyme).

For C6S quantification, resuspended cells were counted in a Neubauer chamber, lysed by ultrasound, and centrifuged at 13,000 rpm to recover the protein extract. Then, 1 mL of DMB was added to 500 μL of supernatant, incubated for 15 min at room temperature, centrifuged for 10 min at 13,000 rpm, and then the supernatant was decanted. Then, 50 μL of 7.5% SDS and 450 μL of purified water were added. The solution was sonicated again and 200 μL was dispensed to a 96 well plate for determination by 595 nm spectrophotometry (EPOCH-2, BioTeK, Swindon ,UK).

#### 2.2.10. In Vivo Biodistribution Study

In vivo biodistribution studies were carried out on wild-type mice (average weight of 60 ± 10 g) supplied by the animal facilities at the University of Santiago de Compostela. The mice were kept in individual cages under controlled humidity (60% ± 5%) and temperature (22 ± 1 °C), with regulated day-night cycles (12/12 h) and fed ad libitum. Experiments were carried out in accordance with the European Union Directive 2010/63/EU for animal experiments and were approved by the Galician Network Committee for Ethics Research (15010/2019/005).

Freeze-dried NLC samples of 300 mg were resuspended in milli-Q water and incubated with 10 µL of DiD (Thermo Fisher Scientific) tetrachloride for 20 min at 37 °C to stain the lipid shell. The free dye was removed by two wash/centrifugation cycles (15,000 rpm at 30 min) and finally resuspended in 1 mL of 0.9% saline solution. A total of 100 µL of the NLC dispersion was administered by rapid intravenous injection into the tail of one of 6 mice, and 24 h after administration, the mice were anesthetized and subsequently euthanized. Finally, the autopsy was performed by removing the brain, liver, kidney, spleen, lung, muscle tissue, cartilage, and kneecap bone. Once removed, a small portion of the organ was fixed in 2% glutaraldehyde for observation by electron microscopy (the preparation method was the same as used for the cells in the internalization studies). Another portion of the organ was submerged in formalin for fixation in paraffin and sliced in a microtome. The slices were incubated in a solution of 4 ’,6-diamidino-2-fenilindol (DAPI, Thermo Fisher Scientific) in order to stain the cell nuclei. The histological section was immobilized with Mowiol for observation with the Leica TCS-SP8 confocal microscope (Leica Microsystem, Buffalo Grove, IL, USA).

## 3. Results and Discussion

### 3.1. Physical Characterization of a Mixture of Lipid Components—Crystallinity

To characterize the crystalline structure of the lipid mixtures, we used DSC and XRD (for composition see Table 2). The mixtures of lipids were obtained under similar conditions as reported previously for NLC preparation. 

To prepare the NLC, a thermosensitive nanogel-core containing elosufase alpha was coated with a mixture of fluid and solid lipids. Obtaining stable NLCs was critical to control the physical properties of the lipid shell. Adequate selection of the lipids avoids stability problems associated with the solid lipid nanoparticles, such as lipid crystallization, phase transition, or gelation tendency. Controlling the solid structure by modulating the melting properties and the lipid transition temperature of the lipid layer is critical to the stability of NLCs. This control could be achieved by the correct selection of solid and liquid lipid components.

The DSC curves obtained for pure solid lipids are shown in Supplementary Figure S1. Pure lipids displayed a clear endothermic signal characteristic of a crystalline structure. Compritol ATO 888 and Dynasan 118 showed higher melting points (T_onset_ > 70 °C) and Dynasan 114 and stearic acid were lower (T_onset_ < 60 °C). The crystalline structure of these solid lipids was confirmed in the XRD patterns (Supplementary Figure S2), showing that the lipid maintained a well-defined crystalline structure.

The thermal behavior of the lipid mixtures is depicted in Supplementary Figure S3. As expected, the DSC traces of the 1/1 w/w mixtures of the solid lipids Compritol ATO 888 y Dynasan 114 (M5) showed a reduction in the melting temperature at 50 °C, which was lower than each pure solid lipid. In addition, a broad endothermic melting band was present, suggesting that a eutectic mixture was formed as a consequence of the treatment used to prepare the samples. DSC curves of the solid–liquid lipid mixtures (M1, M2, M3, and M6) showed an intense reduction in the T_onset_ (20–40 °C) and a significant decrease in the heat flow, indicative of an important loss of crystallinity. Additionally, the incorporation of cholesterol (Supplementary Figure S3) in the mixture also led to a decrease in the T_onset_ and the crystallinity by acting as a plasticizing agent. The reduction in crystallinity was confirmed by the XRD patterns (Supplementary Figure S4). This kind of modification in the thermal behavior of mixtures of solid lipids and liquid lipids was described by other authors [38,39] and may contribute to the stabilization of NLCs.

### 3.2. Preparation and Characterization of Elosulfase Alfa-Loaded NLCs

According to the results obtained from crystallization study using DSC and XRD, the composition of the mixture of solid and liquid lipids selected for the preparation of NLCs is shown in Table 1. The mean size of NLCs was 170 ± 28 nm with a polydispersity of 0.18 and a ζ potential of −29 mV. Unlike previous studies conducted with lipid nanoparticles of stearic acid [40,41] , neither aggregation nor an increase in size were observed in NLCs during storage at 4 °C for three days. Therefore, the NLCs showed better physical stability in suspension, and were easily handled during the experiments. In addition, the effect of the amount of elosulfase alfa added to the preparation medium on the NLC size was studied (Table 3), and no significant changes in size were observed. 

A PEGylated surface on NLC was prepared by adding a PEG-derivative (of d-α tocoferil-polyethylene glycol 1000) during NLC formation. Surface modification of NLCs is a usual strategy not only to reduce protein adsorption, but to increase circulation time once injected by avoiding unspecific phagocytic clearance. To determine the influence of the degree of PEGylation on the morphology, different concentrations of the pegylating agent in the fabrication of NLC (0.1, 0.2, and 0.3 mg/mL) were investigated. NLC synthesis was carried out by maintaining the concentrations of the other components, including the enzyme, and the size and surface charge were determined (Table 4). A small increase in size and a small reduction in surface charge were observed, probably due to the formation of an external PEG layer coating the NLCs.

**Figure 1:**
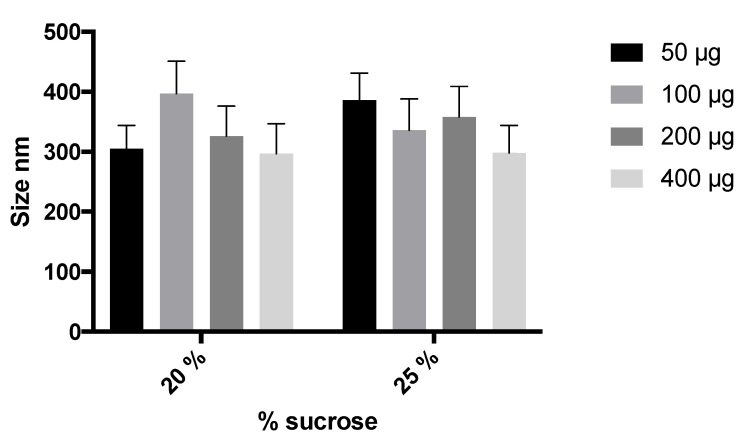
Effect of the percentage of sucrose and enzyme loading on NLC size.

To improve stability and facilitate the manipulation of the NLCs during storage, the NLC swere freeze-dried to obtain a solid dispersible powder. To guarantee the stability and redispersion of lyophilized NLCs, the incorporation of several cryoprotectants was studied (glucose, trehalose, mannitol, and sucrose). After resuspension, glucose (5% and 10%) and mannitol (15%) resulted in aggregated systems with high particle sizes (688.4, 620.2, and 1012 nm, respectively) and polydispersion (>0.7). Trehalose and sucrose at 10% and 20% produced smaller sizes (<500) for particles and better polydispersion (0.5–0.6). Sucrose as a cryoprotectant (20%) led to more suitable NLC characteristics after freeze-drying (334 nm, PdI of 0.5). Also, the effects of 20% or 25% sucrose and the elosulfase alfa-loading on the NLC size were studied. As shown in Figure 1, no significant difference was observed regarding the final particle size. On the basis of these results, 20% sucrose was selected to prepare the freeze-dried NLC formulation. These results were in agreement with other authors [40] that studied the stability of Arylsulfatase A and Arylsulfatase B in lyophilized liposomes, where sucrose was demonstrated to be the most appropriate cryoprotectant.

The final NLC formulation was shown to be of an adequate size range before and after the freeze-dried process to be injected intravenously. Additionally, it was described that nanoparticles ranging between 100 and 300 nm are well-suited to avoid rapid blood circulation elimination and renal clearance, and that soy NLCs have the potential to improve the permanence of the delivery system for longer circulating times.

**Figure 2:**
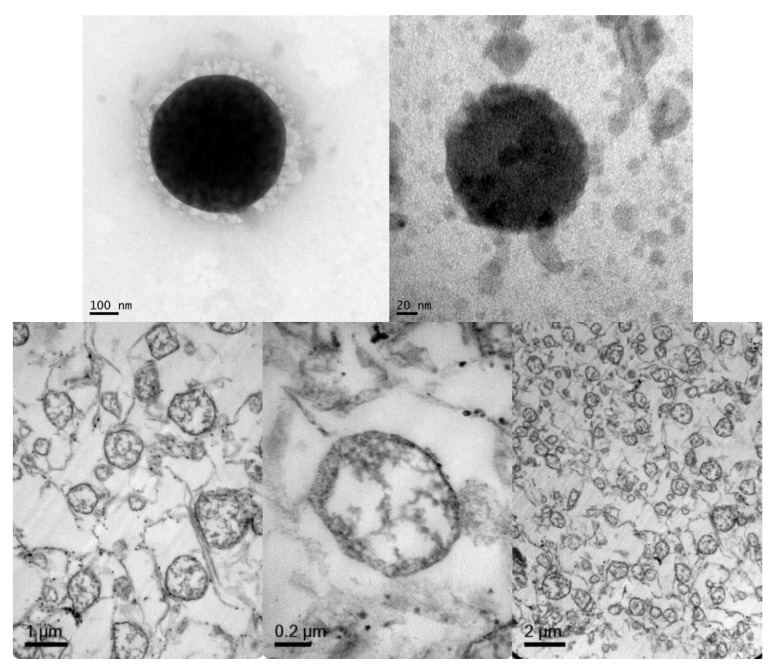
Representative TEM microphotographs of an NLC fixed with phosphor-tungstic acid (**top**) and sectioned with a microtome after lipid staining with osmium tetroxide and uranyl acetate (**bottom**).

Regarding NLC morphology and structure, TEM microphotographs were obtained to visualize NLCs directly (Figure 2, top images) and after lipid staining (Figure 2 bottom). TEM images of the NLCs prepared according to the composition of Table 1 showed particles with spherical shapes with sizes that were consistent with the dynamic light scattering (DLS) measurements. After lipid staining and microtome sectioning of NLCs, a non-lipid core corresponding to the thermosensitive hydrogel core was observed, as well as the lipid-forming shell structure.

**Figure 3:**
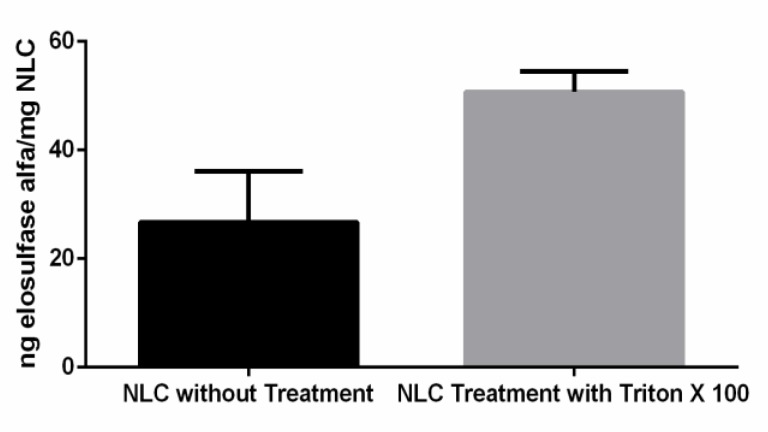
Activity of elosulfase alfa immobilized in the NLC before and after the complete disintegration of particles.

One important aspect to consider is the capacity of the NLC to immobilize the elosulfase alfa and to preserve its enzyme activity and, subsequently, its biological efficacy. The enzyme activity of the elosulfase-NLC system was investigated before and after disintegration of the NLC and the complete release of the enzyme (Figure 3). The results of the enzyme assays confirmed that the enzyme was incorporated into the lipid systems, remaining active after the manufacturing process. The activity observed in intact NLCs indicated that the substrate used for the determination of enzyme activity accessed the immobilized enzyme. These data suggest that a portion of the enzyme was immobilized in the external layer of the NLC, where it was easily accessed by the substrate. The breakage of the NLC led to a significant increase in activity (unpaired two-tailed *t*-test, α > 0.05) as a consequence of the complete release of the enzyme from the internal core of the NLC, to which the substrate had more restricted access.

### 3.3. Plasma Stability of Elosulfase Alfa-NLC

**Figure 4:**
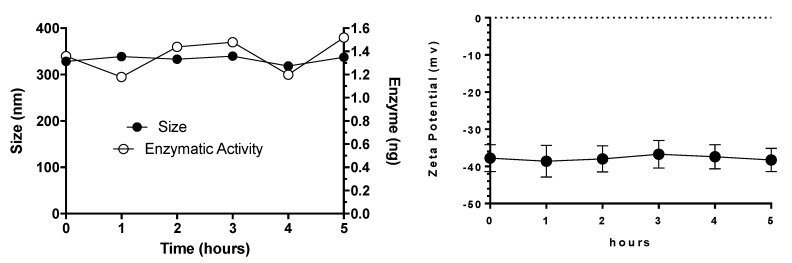
Effect of plasma on elosulfase alfa-NLC stability (size and ζ potential).

Some of the major challenges for the systemic delivery of any drug carrier include stability in the bloodstream and degradation or clearance by the reticuloendothelial system. Since NLCs are administered parenterally, the stability in the presence of plasma proteins plays a key role to perform its biological activity. To investigate NLC behavior in the presence of different proteins, elosulfase alfa-NLCs were dispersed in human plasma and the changes in size, surface charge, and enzyme activity were evaluated (Figure 4). The results demonstrated that the NLC system was stable after 48 h in the plasma environment and that no aggregation or changes in superficial charge and enzyme activity were observed. Additionally, the determination of elosulfase alfa activity after 1, 3, 6, 24, and 48 h of incubation showed that 100% of the enzyme loaded in the NLC mantained its activity.

### 3.4. Cellular Internalization of Elosulfase Alfa-NLC (TC28a2 Chondrocytes)

**Figure 5:**
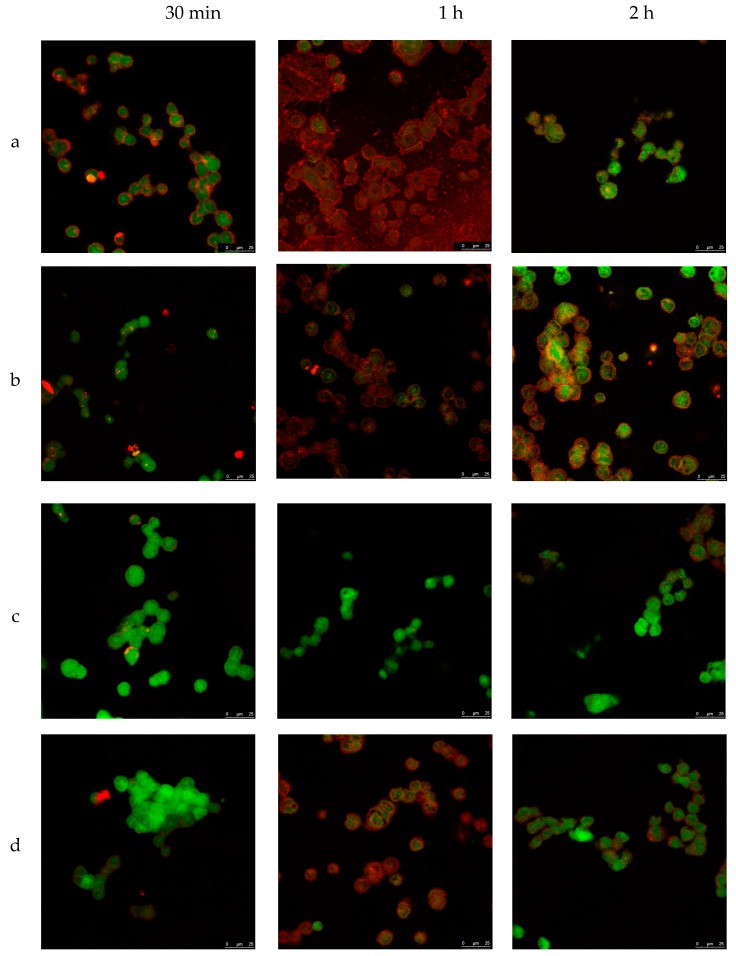
Microscopy confocal images of TC28a2 chondrocyte cells (green) incubated with enzyme-NLC (red) at 37 °C. (**a**) 200 mg/mL, (**b**) 100 mg/mL, (**c**) 50 mg/mL, and (**d**) 25 mg/mL.

To investigate the internalization ability and localization of NLCs in a cell, TC28a2 chondrocytes were co-incubated with NLC in different conditions. The process was studied using confocal microscopy with different fluorophores (see Methods section). To obtain the pictures, the average plane of the sequence of images obtained by superimposition was taken at different wavelengths. Thus, TC28a2 cells were exposed to various enzyme-loaded NLC concentrations for different times and temperatures. Figure 5 shows the confocal images of cells incubated at 37 °C with different concentrations of NLC from 30 min to 2 h. First, the untreated cells (no NLC and no fluorochrome) did not exhibit fluorescence (data not shown), indicating that the cells did not exhibit inherent fluorescence. Second, in all of the analyzed images, the NLCs were detected inside the cells after 1 h of incubation, indicating that internalization of NLCs occurs regardless of NLC concentration in the cell medium.

**Figure 6:**
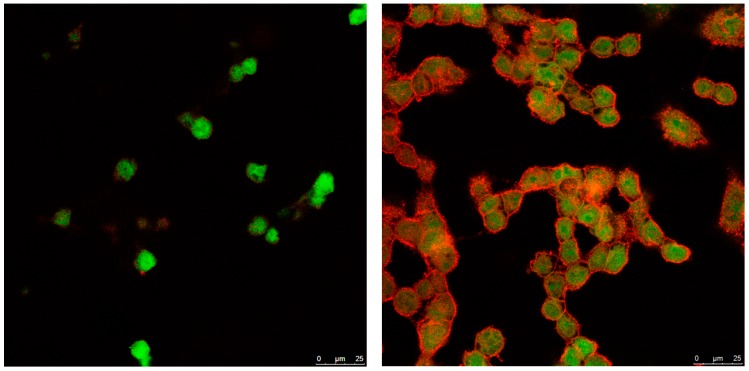
Microscopy confocal images of TC28a2 chondrocyte cells (**green**) incubated with 50 mg/mL of enzyme-NLC (**red**) at 4 °C. **Left**: 30 min; **right**: 1 h.

To gain more insight into the internalization mechanism of NLCs, TC28a2 cells were incubated at a temperature of 4 °C with a low NLC concentration of 50 mg/mL to inhibit the mechanisms of receptor-mediated internalization. The images (Figure 6) showed a negligible internalization of NLC, suggesting that NLCs were internalized mainly through active mechanisms, probably clathrin-mediated endocytosis, an energy-dependent process for entry into the cells.

Hughes et al. [39] studied the internalization of α-galactosidase immobilized in dyed DID-liposomes using HIDC-1 cells and fluorescent confocal microscopy. They observed that after 3 h of incubation at 37 °C, liposomes were found inside the cells, but internalization did not occur at low temperatures (17 °C). Using Lysotracker^TM^ staining, we confirmed the location of the liposomes into the lysosomes.

**Figure 7:**
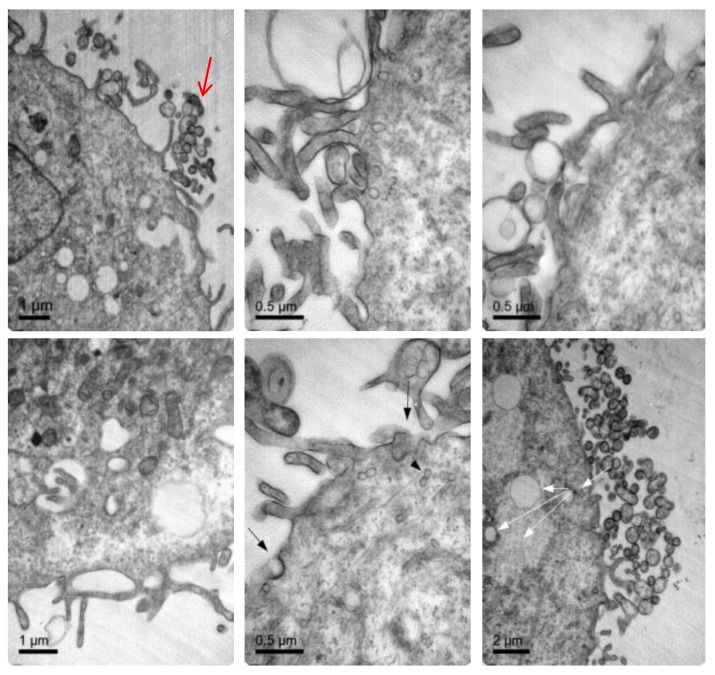
TEM images illustrating elosulfase alfa-loaded NLC internalized into TC28a2 chondrocytes during co-incubation.

**Figure 8:**
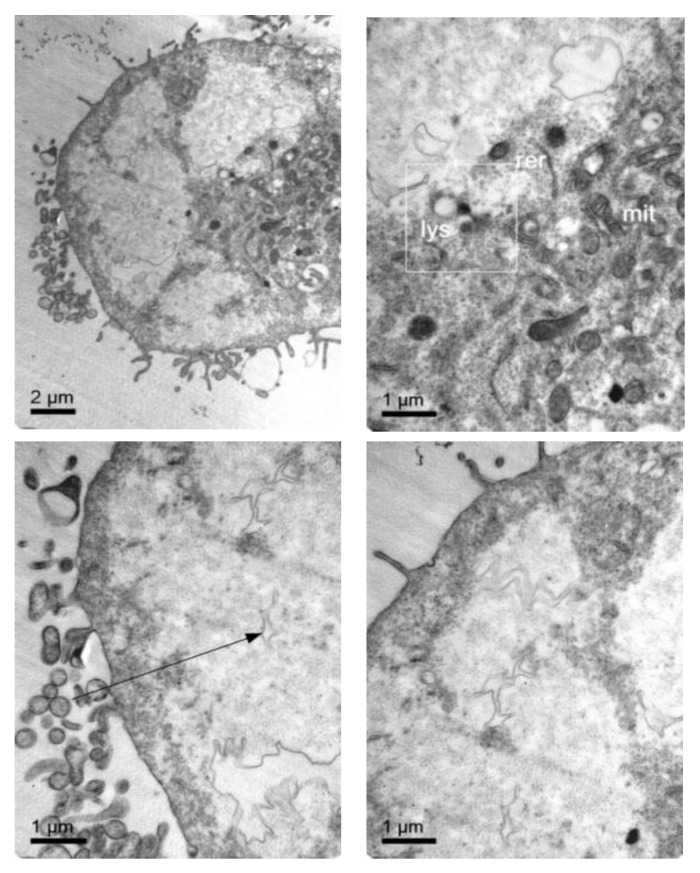
TEM images of the TC28a2 chondrocytes incubated with enzyme-loaded NLCs. The formation of large phagosomes after NLC incubation was observed. Additionally, details regarding the rest of the lipid components after NLC digestion were ascertained.

To further confirm that NLCs were internalized, transmission electron microscopy (TEM) was performed. Figure 7 shows representative TEM images of the TC28a2 chondrocytes after co-incubation of the NLCs. Micrographs showed the interaction of the NLCs with chondrocytes and their internalization. NLCs were distinguished from the rest of the cellular components due to the lipid NLC components staining darker. Thus, it was possible to observe some of the NLCs located around the cell surface interacting with the irregular microvilli of the chondrocytes. These interactions led to the formation of endocytic vesicles around the NLCs that allowed for their internalization. Micrographs clearly showed the formation of phagosomes containing NLCs within the cells (white arrows). Figure 8 shows significant changes in the ultrastructure of the chondrocytes after the internalization of NLCs, where large phagosomes were observed and the fusion of phagosomes with lysosomes to form phagolysosomes was also observed. It should be noted that the cell membranes and organelles remained intact without alterations in their structure and that no alterations of other subcellular organelles (nucleus, mitochondria, etc.) in the cells were detected. Finally, as a consequence of the lipid degradation process in lysosomes, remnants of the lipid components of the NLCs inside the cells were observed.

Once NLC internalization in the chondrocytes was verified, the functionality of elosulfase alfa loaded in NLC was further studied to test whether enzyme activity was maintained inside the cells. With this aim, TC28a2 chondrocytes cells were co-incubated in medium containing immobilized enzyme in the NLC formulation. Cell samples were treated to identify the enzyme by electrophoresis techniques and to quantify the cellular GALNS activity by MALDI-TOF analysis.

**Figure 9:**
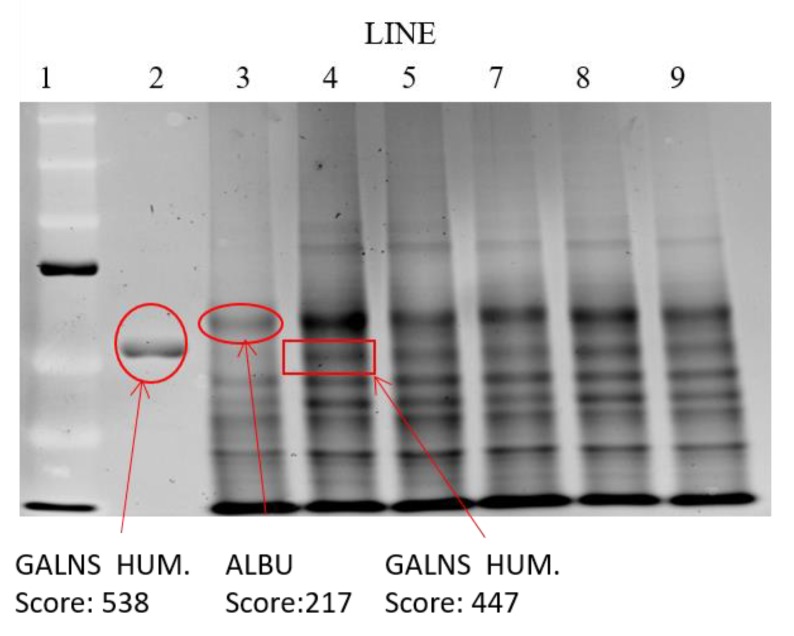
Electrophoretic mobility shift assay of the extracts of cells after co-incubation with elosulfase alfa-NLC. Line 1—molecular weight standards; line 2—elosulfase alfa; Line 3—untreated chondrocytes; Line 4—chondrocytes with elosulfase alfa-NLC.

**Figure 10:**
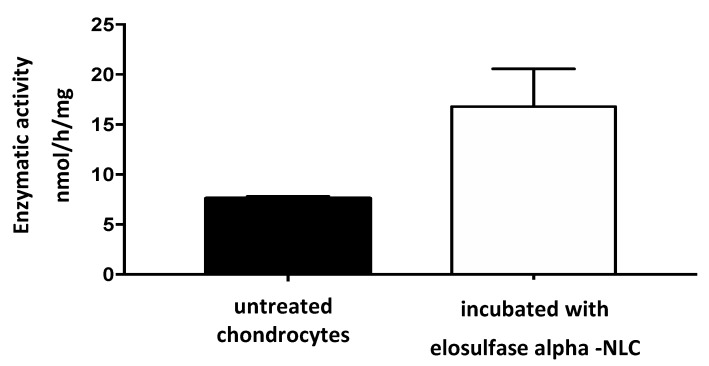
N-acetylgalactosamine 6-sulphatase (GALNS) enzyme activity in untreated chondrocytes and chondrocytes incubated with elosulfase alpha-NLC, quantified by MALDI-TOF analysis.

The results of the analysis of the electrophoretic bands (Figure 9) confirmed the absence of recombinant enzyme in the untreated chondrocytes. However, in the cells treated with the immobilized enzyme in NLCs, an elosulfase alfa band was observed, indicating the incorporation of the enzyme into the chondrocytes. The endogenous cellular GALNS (N-acetylgalactosamine 6-sulphatase) activity in these healthy chondrocytes is shown in Figure 10. A significant increase in the activity of the cells treated with the immobilized recombinant enzyme was observed. This confirmed that the enzyme encapsulated in the NLC reached the lysosomes, where enzyme activity was preserved.

### 3.5. Cellular Internalization of Elosulfase Alfa-NLC and Enzyme Cellular Release in Pathological Fibroblasts from MPS IVA Patients

**Figure 11:**
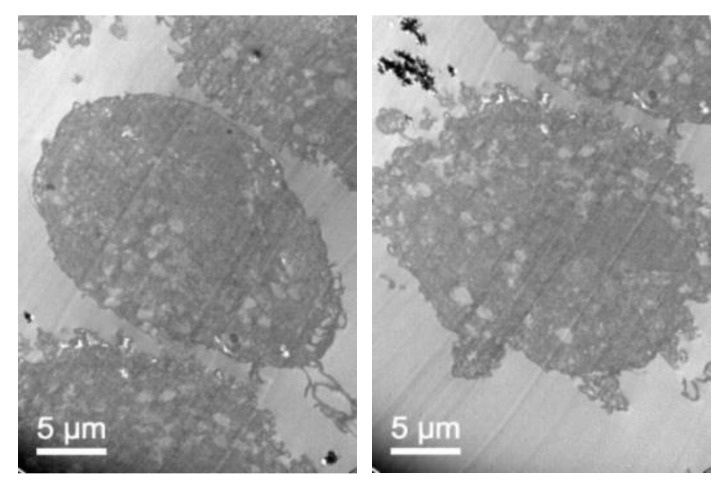
TEM images of pathological fibroblasts from mucopolysaccharoidosis IVA (MPS IVA) patients.

To confirm that NLC with loaded elosulfase alfa were taken up b fibroblasts and to determine the intracellular distribution of the NLC inside the cells, the observation of fibroblasts upon co-incubation with NLC suspension was investigated by TEM. Figure 11 shows TEM images of fibroblasts from donor patients suffering from MPS IVA. To obtain the cells images, samples were stained using Osmium tetraoxide. Pathological fibroblasts presented lighter colored deposits formed by the accumulation of GAG cell clusters due to lysosomal enzyme deficiency.

**Figure 12:**
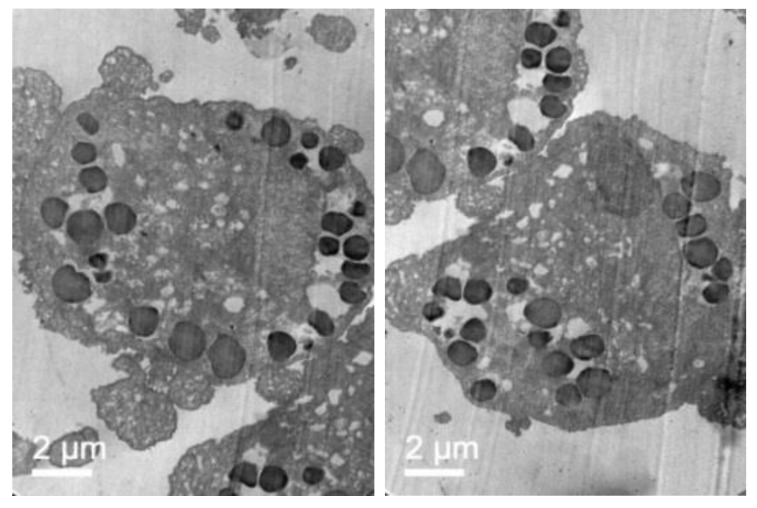
TEM images of pathological fibroblasts incubated with elosulfase alfa-NLC for 3 h.

After fibroblast co-incubation with free enzyme, no appreciable change was observed, but after co-incubation with the elosulfase alfa-NLC suspension (Figure 12), GAG deposit appearance changed significantly. The light grey GAG deposits turned black and increased in size as a consequence of the accumulation of lipid material of the NLCs in lysosomal deposits after cellular internalization. Consequently, TEM images suggested that enzyme-loaded NLCs were internalized in cells accumulating lysosomal deposits. Also, another important finding of this study is the fact that no sign of toxicity of NLC was found at the ultrastructural level of cells over 24 hours. No degeneration of the cell organelles or nucleus was visualized over the incubation time, despite of the substantial number of particles present within the fibroblasts.

**Figure 13:**
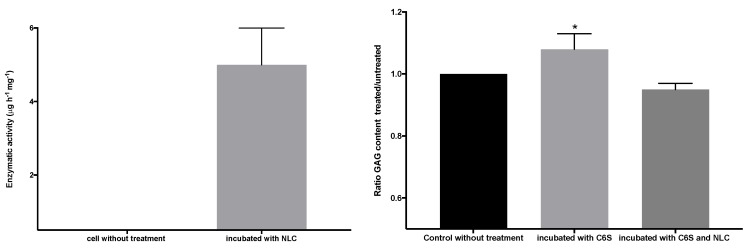
GALNS activity (left graph) and reduction of the glycosaminoglycan (GAG) lysosomal deposits of pathological fibroblasts before and after incubation in a suspension of elosulfase alfa-NLC.

Figure 13 shows the GALNS enzyme activity (left graph) of the pathological fibroblasts before and after incubation in a suspension of elosulfase alfa-NLC. Untreated pathological cells did not show GALNS activity, but the cells treated with elosulfase alfa-NLC significantly increased in activity, indicating internalization and liberation of the enzyme in the lysosomes from the NLC. To confirm the activity of the elosulfase alfa-NLC-treated fibroblasts, the cells were incubated in a C6S solution to promote GAG accumulation inside the lysosomes. The concentration of GAGs in the deposits increased in comparison to the untreated cells (see Figure 13 right). The fibroblasts incubated in the C6S solution with elosulfase alfa-NLC showed significantly reduced GAG contents in the deposits, indicating recuperation of GALNS activity in treated cells. Thus, these results confirmed that the immobilized elosulfase alfa was internalized into the lysosomes, and the enzyme release from NLCs in the lysosomal deposits maintained the GALNS activity. 

### 3.6. Cell Viability Test upon Co-Incubation with NLC

**Figure 14:**
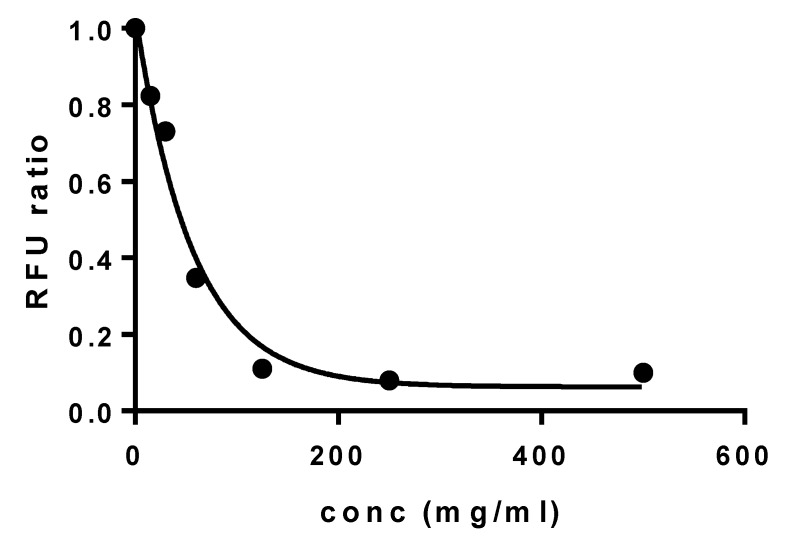
Cell viability of human primary fibroblasts incubated with elosulfase alpha-NLC for 24 h.

The cytocompatibility of elosulfase alpha-loaded NLC was evaluated using a primary culture of human fibroblasts via the AlarmarBlue^®^ test. The results of the fluorescence unit ratio obtained at different concentrations of the formulation are shown in Figure 14.

Results indicated a calculated dosis letal 50 (DL50) value of 56.5 mg/mL. This value was higher than other authors observed for safe auxiliary pharmaceutical substances, such as 2-hydroxypropyl-β-cyclodextrin (DL50 18 m/mL in primary keratocytes, Fernández-Ferreiro A et al. [42]) or Polyethylene glycol 1000 or 2000 (DL50 36.2 mg/mL and 38.2 mg/mL, respectively, in human cervical cancer cells (HeLa) and 22.5 mg/mL and 28.7 mg/mL in fibroblasts derived from mice (L929), Guoqiang Liu et al. [43]). Therefore, these data suggested that the viability of human fibroblasts was not negatively affected during co-incubation with elosulfase alpha-loaded NLCs, confirming the cellular compatibility of NLCs with human cells when compared with controls.

### 3.7. In Vivo NLC Distribution Studies

**Figure 15:**
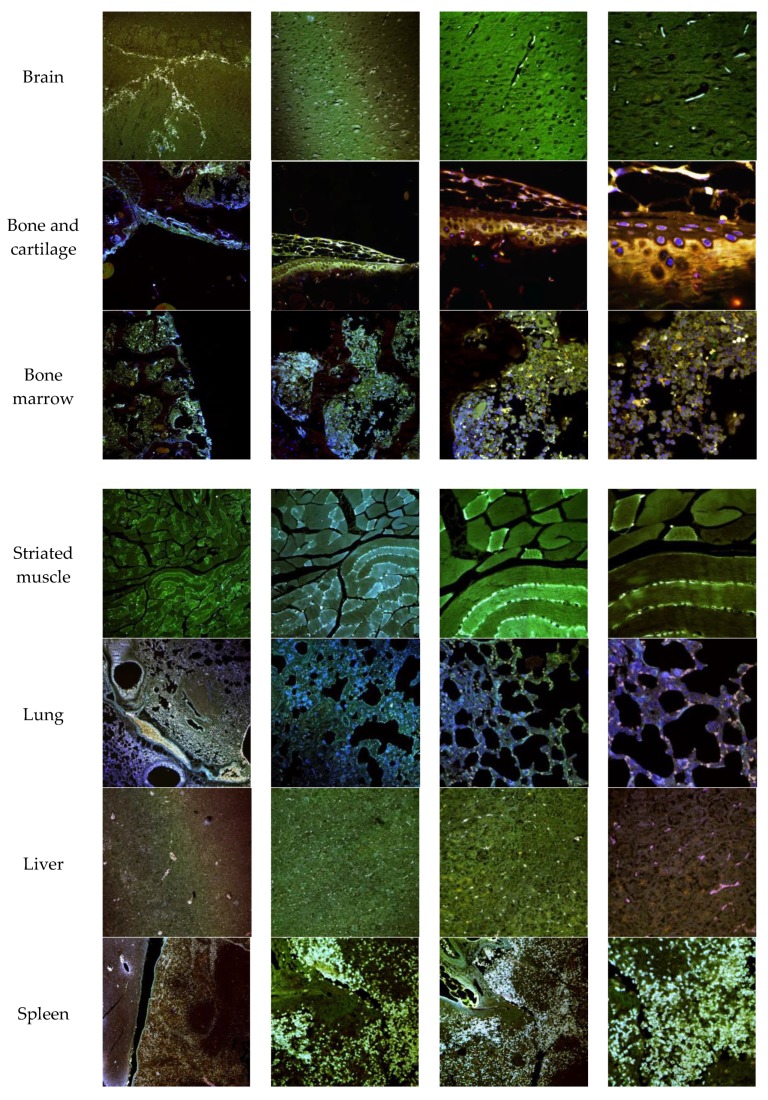

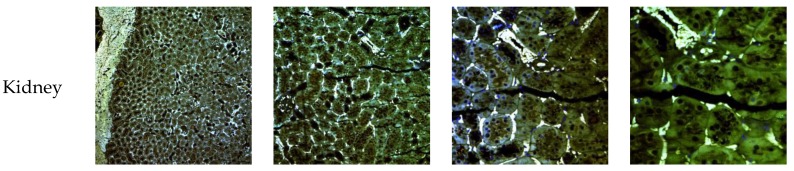
Confocal microscopy images of mice organs 24 h after intravenous administration of elosulfase alpha-NLC stained with DiD. From left to right: Confocal images at 10×, 20×, 40×, and 63×.

**Figure 16:**
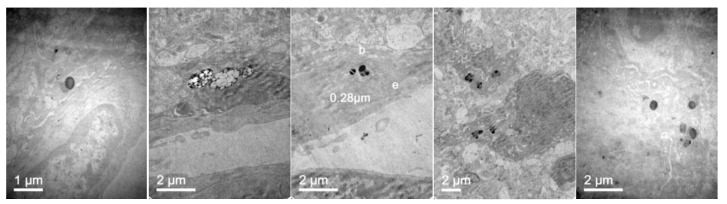
TEM images of the brain in mice 24 h after administration of elosulfase alpha-NLC passing blood-brain barrier (BBB) (**three left images**), in the interior of the astrocytes (**fourth image**), and neurons (**fifth image**).

**Figure 17:**
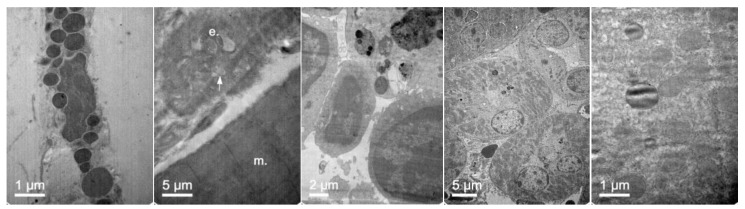
TEM images of (**left to right**) fibroblasts, vascular endothelia between muscle fibers, spleen macrophages, renal tubular cells, and hepatocytes of mice 24 h after administration of elosulfase alpha-NLC.

The biodistribution of enzyme-loaded NLCs stained with DiD was studied after intravenous administration in mice. Mice were euthanatized 24 h after administration and dissected for organ retrieval. Confocal microscopy and TEM images at several magnifications were obtained to locate NLCs in the tissues. Figure 15 shows the confocal microscopy images with details of the tissues. Figure 16 and Figure 17 show the TEM images.

Confocal images showed that NLCs were distributed in all the studied tissues. As expected, the highest concentrations of NLC were found in the best-irrigated tissues, such as the spleen, liver, and lung. However, NLCs were also found in less accessible tissues, such as brain, cartilage, and bone, showing the excellent distribution of NLCs and the capacity to cross the blood-brain barrier (BBB) and to access poorly irrigated tissues. Figure 16 shows the TEM images of brain tissue after NLC treatment. NLCs crossed the BBB and were observed in brain cells, such as astrocytes and neurons. This is significant because, although this is not the case with MPS IVA, some lysosomal diseases have neurological complications. Conventional ERT cannot cross the BBB, so no activity is effected in the brain unless the enzyme is administered directly into the encephalic cavity. Therefore, our system shows the potential for enzyme use in LSDs that cause neurological impairment.

In addition, Figure 17 shows that NLCs can reach and be internalized by cells of different tissues. The capacity of these lipid systems to reach poorly irrigated tissues, such as bone and cartilage, is of special interest. These tissues are one of the main targets for treatment of MPS IVA where the free enzyme cannot be easily distributed.

## 4. Conclusions

In this work, we developed a cytocompatible formulation based on solid lipid carrier systems to deliver the enzyme efficiently to target tissues and successively preserve its activity inside the target cells, chondrocytes. A unique PEGylated nanostructured lipid carrier with a gel core was invented by fast-double emulsification (O/W/O) and low temperature-solidification techniques to encapsulate the enzyme. In vitro data obtained by the co-incubation of elosulfase-alfa-loaded NLC showed cellular uptake and accumulation inside phagolysosomes in human chondrocytes and pathological fibroblasts. 

The in vivo biodistribution study in mice shows the capacity of the NLC to reach less irrigated and less accessible tissues, such as the brain, cartilage, and/or bone, which are important target tissues in LSDs. These findings provide increased insight into enzyme delivery by nanostructured lipid carriers as potential candidates for ERT. Future studies should be undertaken in diseased animal models to further investigate in vivo efficacy and safety.

## Figures and Tables

**Table 1:** Main composition of the nanostructured lipid carrier (NLC) used for elosulfase alfa encapsulation.

**Components (gel core)**	**% in Formulation (*v/v*)**
Kolliphor^®^ P407	60
Elosulfase alfa	40
**Components (lipid coating)**	**% in Formulation (*w/v*)**
Glyceril dibehenate	8.24
Trymiristin	8.24
Triestearin	8.24
Cholesterol	3.84
Olive oil	32.96
Caprylic/capric tryglicerides	27.47
Soy lecitin	10.98

**Table 2:** Lipid mixtures used to characterize the physical structure of the lipid-forming shells of NLCs.

Lipid	M1	M2	M3	M4	M5	M6
Compritol ATO 888	25.0 mg	75.0 mg	-	-	91.5 mg	37.0 mg
Dynassan 114	25.0 mg	-	37.5 mg	-	91.5 mg	25.0 mg
Dynassan 118	25.0 mg	-	37.5 mg	-	-	25.0 mg
Cholesterol	12.0 mg	12.0 mg	12.0 mg	-	-	-
Olive Oil	100.0 mg	100.0 mg	100.0 mg	100.0 mg	-	100.0 mg
Miglyol	33.0 mg	33.0 mg	33.0 mg	33.0 mg	-	33.0 mg

**Table 3:** Influence of the quantity of elosulfase alfa added to the preparation media on the size of the NLCs.

Enzyme Added (μg)	Mean Size (nm)	PdI
50	169.0	0.241
100	174.7	0.306
200	173.0	0.270
400	175.7	0.150
500	192.1	0.207

**Table 4:** Influence of the PEGylant agent concentration on the size and potential ζ of the NLCs.

d-α-tocopheryl-polyethylene Glycol 1000 Succinate Concentration (mg/mL)	Mean Size (nm)	PdI	Potential ζ (mv)
0.1	200.8 ± 15	0.236 ± 0.04	−18.7 ± 0.3
0.2	214.5 ± 12	0.158 ± 0.03	−16.3 ± 0.3
0.3	195.8 ± 10	0.164 ± 0.02	−14.2 ± 0.2

## Supplementary Materials

The following are available online at https://www.mdpi.com/1999-4923/11/10/522/s1, Figure S1. DSC curves of the solid lipids used to prepare the lipid shell of the NLC; Figure S2. XRD patterns of solid lipid after you process in same conditions that used in NLC production; Figure S3. DSC curves of the mixtures of the solid and liquid lipids used to prepare the NLC lipid shell; Figure S4. XRD patterns of mixtures of lipid and solid lipid used to prepare the NLC lipid shell.
