# Supplementary material for: Enzyme-Loaded Gel Core Nanostructured Lipid Carriers to Improve Treatment of Lysosomal Storage Diseases: Formulation and In Vitro Cellular Studies of Elosulfase Alfa-Loaded Systems
Source: Pharmaceutics. 2019 Oct 11;11(10):522. doi: 10.3390/pharmaceutics11100522 (PMC6835858; doi:10.3390/pharmaceutics11100522)
Supplement: Supplementary file 1 [file pharmaceutics-11-00522-s001.pdf]

pharmaceutics

Supplementary Materials: Enzyme-Loaded Gel Core Nanostructured Lipid Carriers to Improve Treatment of Lysosomal Storage Diseases: Formulation and In Vitro Cellular Studies of Elosulfase Alfa-Loaded Systems

J. Víctor Álvarez, Carolina Herrero Filgueira, Alexandre de la Fuente González, Cristóbal Colón Mejeras, Andrés Beiras Iglesias, Shunji Tomatsu, José Blanco Méndez, Asteria Luzardo Álvarez, María Luz Couce and Francisco J Otero Espinar

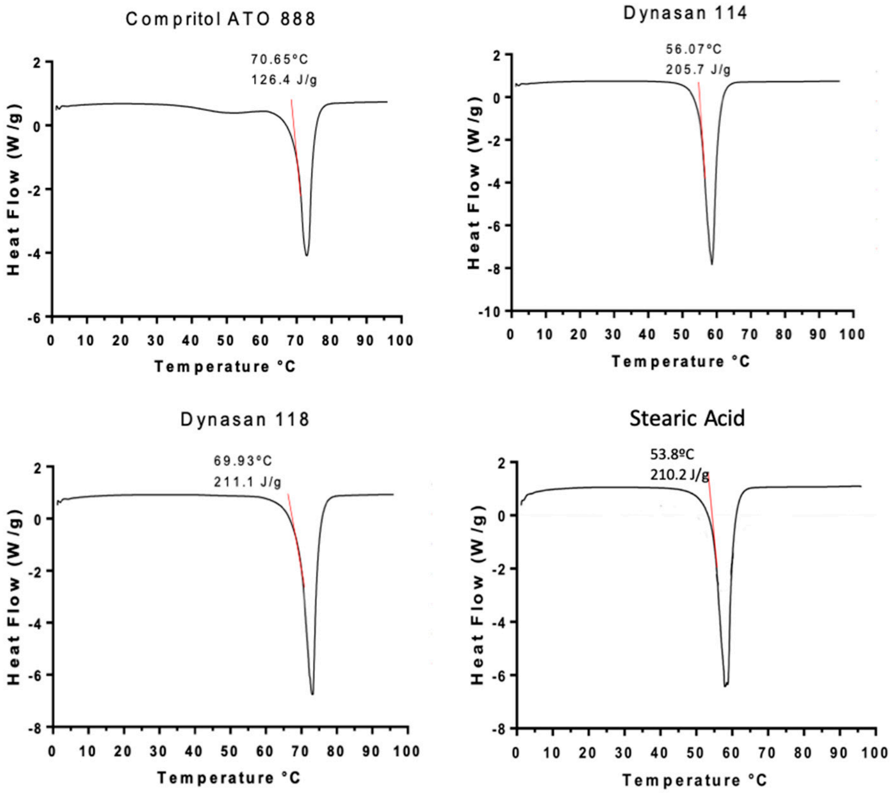

Figure S1. DSC curves of the solid lipids used to prepare the lipid shell of the NLC.

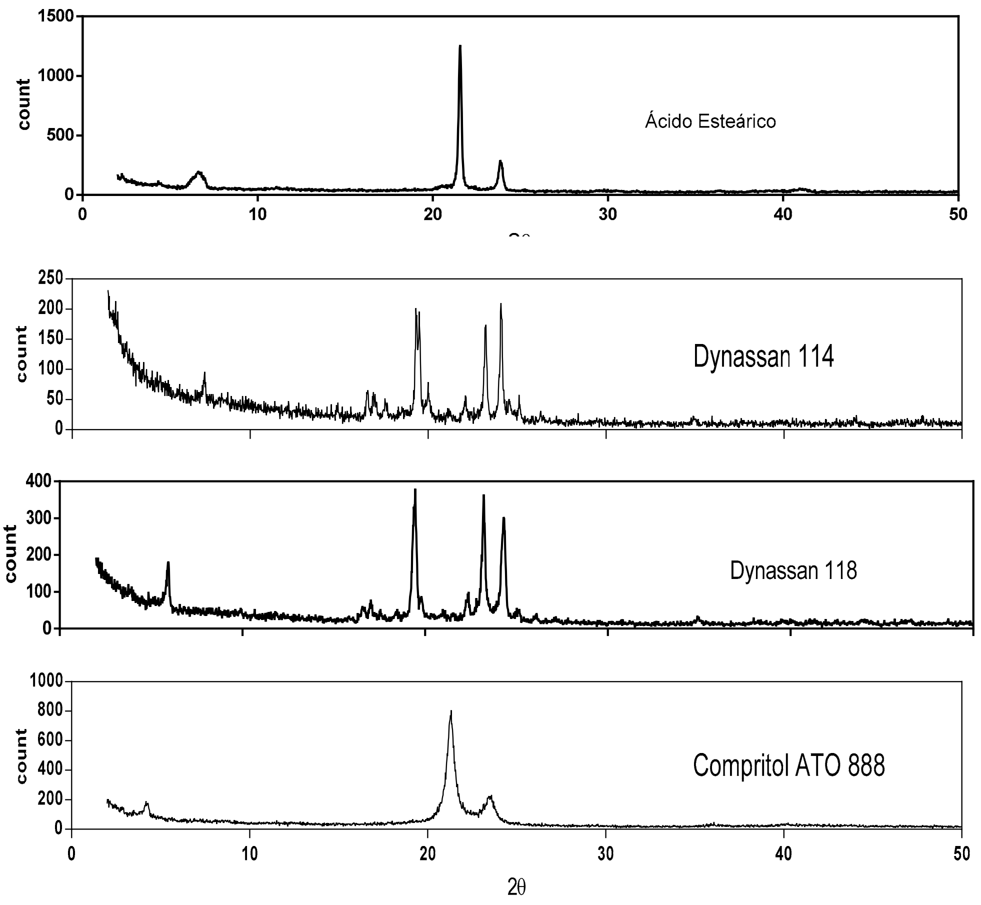

Figure S2. XRD patterns of solid lipid after you process in same conditions that used in NLC production.

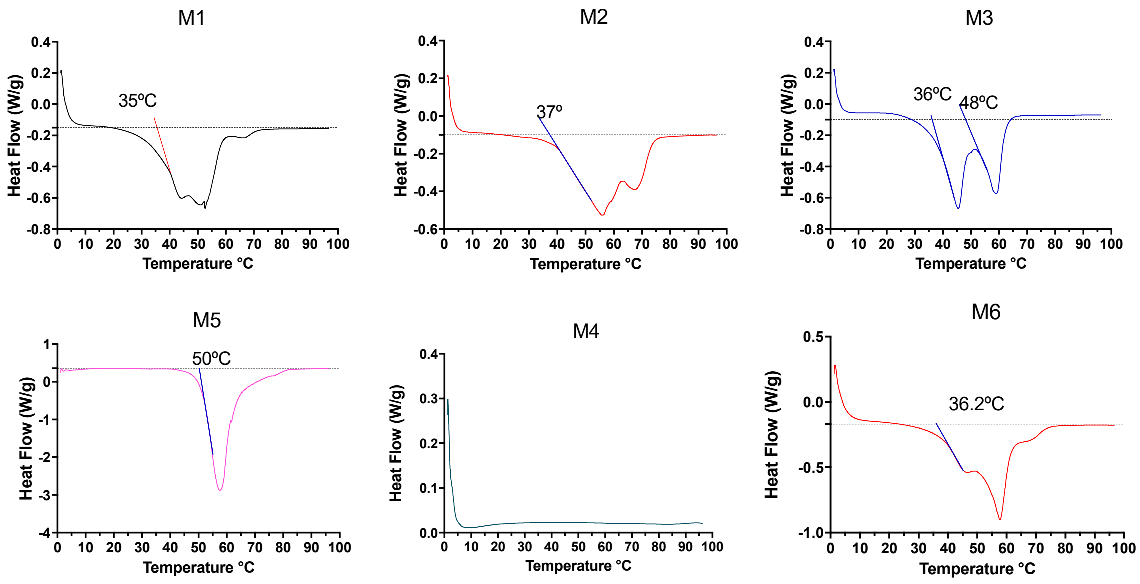

Figure S3. DSC curves of the mixtures of the solid and liquid lipids used to prepare the NLC lipid shell.

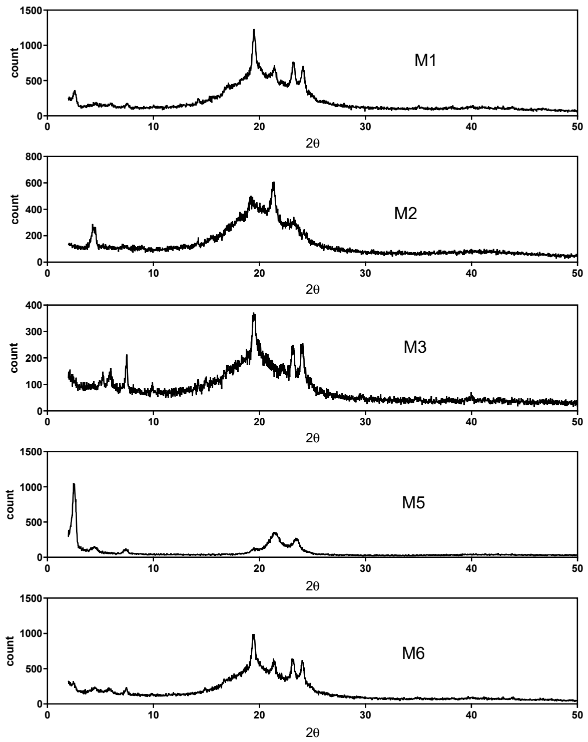

Figure S4. XRD patterns of mixtures of lipid and solid lipid used to prepare the NLC lipid shell.
